# Extended-interval Dosing of Gentamicin for Treatment of Neonatal Sepsis in Developed and Developing Countries

**Published:** 2008-06

**Authors:** Gary L. Darmstadt, Mary Miller-Bell, Maneesh Batra, Paul Law, Kiely Law

**Affiliations:** ^1^Department of International Health, Bloomberg School of Public Health, Johns Hopkins University, Baltimore, MD 21205, USA; ^2^Department of Pharmacology, Duke University, Durham, NC, USA; ^3^Department of Pediatrics, University of Washington School of Medicine, Seattle, WA, USA; ^4^Department of Informatics, Kennedy Krieger Institute, Baltimore, MD, USA

**Keywords:** Developing countries, Drug therapy, Gentamicin, Infant, Newborn, Pharmacokinetics, Review literature, Sepsis

## Abstract

Serious bacterial infections are the single most important cause of neonatal mortality in developing countries. Case-fatality rates for neonatal sepsis in developing countries are high, partly because of inadequate administration of necessary antibiotics. For the treatment of neonatal sepsis in resource-poor, high-mortality settings in developing countries where most neonatal deaths occur, simplified treatment regimens are needed. Recommended therapy for neonatal sepsis includes gentamicin, a parenteral aminoglycoside antibiotic, which has excellent activity against gram-negative bacteria, in combination with an antimicrobial with potent gram-positive activity. Traditionally, gentamicin has been administered 2–3 times daily. However, recent evidence suggests that extended-interval (i.e. ≥24 hours) dosing may be applicable to neonates. This review examines the available data from randomized and non-randomized studies of extended-interval dosing of gentamicin in neonates from both developed and developing countries. Available data on the use of gentamicin among neonates suggest that extended dosing intervals and higher doses (>4 mg/kg) confer a favourable pharmacokinetic profile, the potential for enhanced clinical efficacy and decreased toxicity at reduced cost. In conclusion, the following simplified weight-based dosing regimen for the treatment of serious neonatal infections in developing countries is recommended: 13.5 mg (absolute dose) every 24 hours for neonates of ≥2,500 g, 10 mg every 24 hours for neonates of 2,000–2,499 g, and 10 mg every 48 hours for neonates of <2,000 g.

## INTRODUCTION

### Global importance of neonatal infections

An estimated four million neonatal deaths occur around the world every year (1). Approximately 99% of these deaths occur in developing countries (1-3). Serious bacterial infections are the single most important cause of morbidity and mortality among newborns (1,4-6). An estimated 20% of all children born in developing countries, or 30 million annually, develop an infection during the neonatal period, and infectious diseases account for 36% of all neonatal deaths (1,7-9). Recent data suggest that approximately one-half of neonatal deaths in high-mortality settings are due to infections (1,4,10).

For the treatment of serious bacterial infections in neonates, the World Health Organization (WHO) recommends intramuscular injections of an aminoglycoside and penicillin antibiotics for at least 10 days (11). The recommendations of WHO were designed for infants aged seven days to two months and include gentamicin dosed at 7.5 mg/kg intramuscularly once daily (11). Gentamicin is a potent aminoglycoside antibiotic with bactericidal activity against gram-negative bacteria. The combination of gentamicin and a penicillin, such as ampicillin, also produces synergistic activity against several principal gram-positive pathogens in neonates. In addition to the use of gentamicin in combination with an injectable penicillin, alternative treatment regimens, such as combining gentamicin with administration of oral antibiotics, including co-trimoxazole, may be life-saving (10). However, until further studies are available which provide evidence for efficacy of oral antibiotic treatment of neonates with suspected sepsis, or perhaps a subset with low-risk indications, every attempt should be made to provide a full course of parenteral antibiotics. Thus, a primary variable in treatment regimens that may potentially be altered to simplify dosing is the duration of the interval between administrations of doses.

The case-fatality rate due to neonatal sepsis in developing countries is estimated at 40%, based largely on data for infants treated in hospitals (9,12). When neonatal infections occur, many deaths can be avoided if the signs are recognized early and the disease is treated promptly and adequately (3,13-15). In rural India and Bangladesh, for example, 66–75% and 88–90% of births, respectively, take place at home, and acceptance of delivery in a health facility by rural women is still minimal (16-21). Since signs of illness due to infections are most likely to manifest while the infant is at home, and families in many societies are reluctant to take newborns outside the home, even when they are ill (20), an important strategy for reducing neonatal mortality will be to improve the ability of caregivers in the family and community to recognize danger signs and to promptly seek care. The ability of first-line health workers to prevent, recognize, and provide initial case management of infectious diseases in the home and community, or at health facilities, will also need to be improved (10,22-24).

For the treatment of neonatal sepsis in resource-poor, high-mortality settings in developing countries where most neonatal deaths occur (1), simplified regimens are needed which ideally would allow for extended-interval dosing of parenteral antibio-tics no more frequently than once a day. Although extended-interval dosing with parenteral antibiotics is desirable in developed countries, it is essential in developing-country community settings. Home-visits by community health workers to administer parenteral antibiotics, or alternatively, visits to the health facility by patients to receive injectable antibiotics generally are not feasible more than once per day. Extended-interval dosing in health facilities could also potentially reduce costs associated with antibiotic treatment, including demands on staff time, reduce demands on logistic and supply systems, and minimize chances for iatrogenic problems associated with antibiotic administration.

In this article, we reviewed the safety and efficacy of extended-interval dosing of gentamicin and the implications for treatment strategies for neonatal sepsis in developing countries. We also recommend a feasible, simplified dosing strategy.

## MATERIALS AND METHODS

We performed a MEDLINE/PubMed search for any published studies of extended-interval dosing of aminoglycoside in the literature in English. We used the following search strategies: (a) aminoglycoside and newborn and dosing, (b) gentamicin and newborn and dosing, (c) treatment and newborn and sepsis, (d) aminoglycoside and dosing and sepsis, and (e) gentamicin and dosing and sepsis. This review includes all the relevant published studies that included information on aminoglycoside, specifically gentamicin, dose, dose interval, serum concentrations, efficacy, and signs of toxicity.

## RESULTS

### Gentamicin pharmacokinetic studies: overview

#### Intramuscular vs intravenous injection

Given the limited access that exists to the supplies and equipment needed to provide intravenous antibiotic therapy in community-based settings and in many health facilities in developing countries, it is often necessary to administer antibiotics by the intramuscular route. Results of pharmacokinetic studies of aminoglycosides in general and gentamicin in particular have demonstrated that the serum concentration-time curves after an intramuscular injection and a 20- to 30-minute intravenous infusion are nearly superimposable (25-34). The six-hour serum concentrations, half-lives, and area-under-the-curve values are also equivalent. Thus, data from studies that used intravenous administration can be extrapolated to settings, such as developing-country peripheral health facility or community settings, where intravenous administration generally is not possible and where the drug often will be delivered by the intramuscular route.

### Pharmacokinetic principles of extended-interval dosing

#### Efficacy

Previously, it was standard of care to deliver gentamicin in multiple doses per day, but in recent years there has been a trend towards administration of higher doses at prolonged intervals. Changes in dosing towards once-daily administration of gentamicin were first evaluated and are now widely implemented in the care of adult patients. Once-daily dosing for neonatal patients is now also being recognized as having many potential benefits (Table 1).

**Table 1:** Advantages of once-daily and extended-interval gentamicin dosing regimens compared to multiple-daily dosing regimens

Higher peak serum levels and higher peak
level: MIC ratio
Prolonged post-antibiotic effect (i.e. prolonged efficacy)
Greater initial bacterial killing
Reduced risk for emergence of resistant strains of bacteria
Sub-toxic drug trough levels maintained for longer periods
Reduced risk for ototoxicity and nephrotoxicity
More cost-effective
Reduced costs for supplies, preparation, and administration of drug
Reduced costs for therapeutic drug monitoring
Reduced costs for managing complications due to drug toxicity

MIC=Minimum inhibitory concentration

Higher doses given at extended intervals allow for more rapid achievement of sufficiently high peak levels of drug to kill susceptible pathogens, while allowing time in-between doses to reach sufficiently low trough levels to avoid toxicity. Gentamicin exhibits a linear relationship between higher peak to minimum inhibitory concentration (MIC) ratio and improved clinical response. The post-antibio-tic effect—or the ability of the drug to continue to suppress bacterial growth even after antibiotic concentrations have fallen below the MIC for the organism—of gentamicin is also concentration-dependent (25,35). Moreover, adaptive resistance is thought to occur after continuous exposure of bacteria to antibiotic concentrations that are less than the MIC.

Current research on administration of gentamicin in neonates further extends these principals to include even higher doses given at intervals longer than 24 hours, such as 36–48 hours (36,37). Since this approach to administration of gentamicin is relatively new, there are few safety data available in large numbers of patients, particularly neonates, and even fewer data from developing-country settings in neonates who received higher doses at prolonged intervals; these data are reviewed below. Data are reviewed separately for neonates in developed- and developing-country settings due to differences in various factors, including newborn care (e.g. fluid administration and monitoring and, thus, hydration status) and health status (e.g. severity of illness at the time of presentation due to delays in seeking, reaching, and receiving appropriate care) (38).

In adult patients, clinical outcome, including mortality due to sepsis or pneumonia (39-42), was shown to depend principally on rapid achievement of therapeutic peak serum levels of drug on the first day of treatment. Mortality from sepsis or pneumonia dropped from 21% to 2.4% depending on whether or not serum gentamicin levels exceeded 5 μg/mL on the first day (40), and in another study, cure rate from bacteraemia was 88% vs 12% depending on whether or not peak serum levels exceeded 5 or 8 μg/mL in patients with sepsis and pneumonia respectively within the first three days (39).

#### Toxicity

The principal adverse effects of gentamicin therapy are renal toxicity which nearly always is reversible and ototoxicity which generally is not reversible. Risk factors for aminoglycoside toxicity include baseline renal impairment, prolonged and repeat courses of administration of gentamicin, and in particular, prolonged serum concentrations above threshold levels, specifically prolonged high trough concentrations that exceed 2 μg/mL for longer than 10 days (25,43). The amount of drug that accumulates in sensitive organs, namely the kidney and inner ear, increases with higher plasma concentrations and longer periods of exposure. Elimination of drug from these organs, on the other hand, occurs more slowly than from plasma and is retarded by high plasma concentrations. Back diffusion from these sensitive organs to the blood, thereby lowering end-organ drug levels, is dependent primarily on the trough rather than the peak concentration of drug in the serum. This accounts for the association between toxicity and high plasma trough concentrations. Once-daily dosing or extended-interval dosing, despite the higher peak concentration, provides a longer period when con serum centrations are below the threshold for toxicity than do multiple-daily dosing regimens, leading to reduced risk for toxicity.

Experience with once-daily dosing regimens strongly suggests that high peak levels of 3 to 4-fold higher than those achieved with multiple-daily dosing regimens, even in excess of 25 μg/mL, do not increase toxicity (25,44-46). In adult patients, enough experience has accumulated with once-daily dosing regimens that peak concentrations are no longer determined routinely. Prolonged dosing intervals with higher doses of drug may minimize the risk of nephrotoxicity because renal cortical uptake of drug appears to be saturable, reaching a plateau despite increasing peak levels of drug. In general, results of studies in animal models have shown that administration of larger, less-frequent doses of aminoglycoside results in lower renal cortical concentrations of drug than found using lower-dose, multiple-daily dosing (47). Similarly, although the relationship between ototoxicity and aminoglycoside dosing regimen or threshold levels for toxici-ty is less well-understood, available data suggest that once-daily dosing of aminoglycosides does not increase the risk of ototoxicity (48). Neonates, especially preterm and sick term infants, have low glomerular filtration rates which accounts for their slower clearance of medications, such as aminoglycosides (49,50). Therefore, extended-interval dosing may be particularly advantageous in neonates by allowing for safer trough concentrations to be achieved.

#### Cost-savings

Extended-interval is a more cost-effective method for treating patients with gentamicin (Table 1) (51,52). There are fewer doses to prepare and administer per day than with multiple-daily dosing. Once-daily dosing saves costs of drug supply, pharmacy and nursing personnel, and costs of therapeutic drug monitoring are reduced because fewer determinations of serum gentamicin concentrations are necessary to monitor for ototoxicity and nephrotoxicity.

### Summary of studies on extended-interval dosing in neonates

Several studies have been published on extended-interval dosing of gentamicin in neonatal patients in both developed (20 studies, Table 2) and developing-country settings (8 studies, Table 3) (26,36,37,51,53-72,76-79). As noted above, we examined the pharmacokinetic parameters in these two groups of patients separately. Experts maintain that the trough levels should be <2 μg/mL (37,60,73), and some now recommend that the trough level should be <1 μg/mL (74). Most experts also recommend that the goal for peak serum levels in neonates should be 5–12 μg/mL (37,60,73). Thus, the literature was reviewed with these target parameters in mind as a guide to appropriate dosing. We present information regarding procedures for monitoring of serum gentamicin concentration for each study included in this review whenever available (Table 2-3). Although monitoring of serum creatinine levels and urine output to assess renal function and toxicity are recommended for patients receiving gentamicin therapy, procedures for such monitoring were not consistently or uniformly followed among the studies included in this review. Furthermore, as serum creatinine levels in neonates vary by gestational age, chronologic age, weight, and maternal renal function, interpretation of these values is complex. As such, we did not include data regarding serum creatinine levels, or potential renal toxicity in Table 2-3.

**Table 2:** Studies on extended-interval gentamicin dosing in neonates in developed countries

Reference	Population	Exclusions	Dose	Monitoring of levels	Mean trough level (μg/mL)	Mean peak level (μg/mL)	Conclusions
Skopnik *et al.* (1992) (57)	Full-term neonates ODD: n=10 TDD: n=10	-<37 weeks -Apgar scores ≤4 (1 minute) or ≤6 (5 minutes) -Birthweight <2,500 g -SCr >85 μg/L -Diuretics	ODD: 4 mg/kg for 24 hours TDD: 2 mg/kg for 12 hours	SGC were obtained 1, 4, 6, 12, or 24 hours after start of infusion	ODD: 0.8 TDD: 1.0 0 patients >2	ODD: 10.9 TDD: 7.4 0 patients <4	ODD higher peaks than TDD
Lopez-Samblas *et al.* (1992) (64)	Study group: n=68 Control group: n=59 (Historical controls)	-BUN >10.7 mmol/L -SCR >106 mcmol/L -Indomethacin treatment	Control: According to attending physician Study: <30 weeks: 3 mg/kg for 24 hours 30–37 weeks: 2.5 mg/kg for 18 hours	Peak: 1 hour after dose Trough: ½ hour or less before dose	Study: 1.4 (90% <2) Control: 2.3 (35% <2)	Study: 6.4 Control: 6.1	EID troughs less likely to be in toxic range
Skopnik *et al.* (1995) (61)	≥37 weeks ODD: n=79 TDD: n=223	-Apgars ≤4 at 1 minute and/or ≤6 at 5 minutes -Abnormal renal function -SGA or LGA infants	ODD: 3.5–4 mg/kg for 24 hours TDD: 2–2.5 mg/kg for 12 hours	Pre: just prior to dose Post: 1 hour after infusion	ODD: 0 paients >2	ODD: 100% >4 1 patient >12	EID safe and effective
Hayani *et al.* (1997) (26)	>34 weeks >2,000 g SCR <1.2 μg/mL ODD: n=11 (IM, n=4) TDD: n=15	-Renal failure -CPR -Shock -Seizures	ODD: 5 mg/kg for 24 hours TDD: 2.5 mg/kg for 12 hours	Pre: 30 minutes before dose Post: 30 minutes after infusion; 60 minutes after IM	ODD: 1.7±0.4 (9% >2) TDD: 1.7±0.5 (40% >2)	ODD: 10.7±2.1 (100% >5; 27% >12) TDD: 6.6±1.3 (13% <5)	ODD resulted in more therapeutic and less toxic SGC
de Alba *et al.* (1998) (53)	>1,200 g SCR <1.2 mcg/mL ODD: n=33 TDD: n=32	-Renal failure -Birth asphyxia	ODD: 5 mg/kg for 24 hours TDD: 2.5 mg/kg for 12 hours	Pre: just prior to administration Post: 2 hours after start of infusion	ODD: 1.4±0.7 TDD: 2.2±1	ODD: 9.5±1.7 TDD: 6.4±1.6	ODD resulted in more therapeutic and less toxic SGC. 2/13 and 1/11 patients in ODD and TDD respectively failed hearing screens
Langlass *et al.* (1999) (54)	>30 weeks ODD: n=74 MDD: n=69	None	ODD: 3.5 mg/kg for 24 hours TDD: 2.5 mg/kg for 12, for 18, or for 24 hours	Pre: 30 minutes before 4th dose Post: 30 minutes after infusion of 3rd dose	ODD: 1.04±0.5 (0 patients >2) MDD: 1.68±0.68 (33% >2)	ODD: 6.98±1.29 MDD: 6.07±1.15	ODD resulted in more therapeutic and less toxic SGC
Logsden *et al.* (1999) (55)	ODD: n=71 No control group	None	3 mg/kg >34 weeks: for 18 hours 26–33 weeks: for 24 hours <26 weeks: for 36 hours	Pre: 30 minutes before dose Post: 30 minutes after infusion	1.4±0.6 (11% >2)	7.8±1.5 (100% >4)	SGC safe and effective
Lundergan *et al.* (1999) (63)	≤7 days of life n=132 courses of gentamicin No control group	None	Load: 5 mg/kg then: ≥2,500 g: 4 mg/kg for 24 hours <2,500 g: 2.5 mg/kg for 24 hours	Pre: just prior to administration of 1st maintenance dose Post: 60 minutes after infusion	0.9±0.2 (0 patients >2)	7.8±1.1 (100% 5–12)	May need fewer SGC monitored than TDD; SGC safe and effective. No significant differences noted on hearing tests (brainstem auditory evoked potentials)
de Alba *et al.* (1998) (53)	>1,200 g SCR <1.2 mcg/mL ODD: n=33 TDD: n=32	-Renal failure -Birth asphyxia	ODD: 5 mg/kg for 24 hours TDD: 2.5 mg/kg for 12 hours	Pre: just prior to administration Post: 2 hours after start of infusion	ODD: 1.4±0.7 TDD: 2.2±1	ODD: 9.5±1.7 TDD: 6.4±1.6	ODD resulted in more therapeutic and less toxic SGC. 2/13 and 1/11 patients in ODD and TDD respectively failed hearing screens
Langlass *et al.* (1999) (54)	>30 weeks ODD: n=74 MDD: n=69	None	ODD: 3.5 mg/kg for 24 hours TDD: 2.5 mg/kg for 12, for 18, or for 24 hours	Pre: 30 minutes before 4th dose Post: 30 minutes after infusion of 3rd dose	ODD: 1.04±0.5 (0 patients >2) MDD: 1.68±0.68 (33% >2)	ODD: 6.98±1.29 MDD: 6.07±1.15	ODD resulted in more therapeutic and less toxic SGC
Logsden *et al.* (1999) (55)	ODD: n=71 No control group	None	3 mg/kg >34 weeks: for 18 hours 26–33 weeks: for 24 hours <26 weeks: for 36 hours	Pre: 30 minutes before dose Post: 30 minutes after infusion	1.4±0.6 (11% >2)	7.8±1.5 (100% >4)	SGC safe and effective
Lundergan *et al.* (1999) (63)	≤7 days of life n=132 courses of gentamicin No control group	None	Load: 5 mg/kg then: ≥2,500 g: 4 mg/kg for 24 hours <2,500 g: 2.5 mg/kg for 24 hours	Pre: just prior to administration of 1st maintenance dose Post: 60 minutes after infusion	0.9±0.2 (0 patients >2)	7.8±1.1 (100% 5–12)	May need fewer SGC monitored than TDD; SGC safe and effective. No significant differences noted on hearing tests (brainstem auditory evoked potentials)
Thureen *et al.* (1999) (51)	≥34 weeks <7 days of life ODD: n=27 TDD: n=28 Also performed cost-effectiveness analyses	-Apgars ≤4 at 1 minute and/or ≤6 at 5 minutes -Decreased urine output -Inotropic support	ODD: 4 mg/kg for 24 hours TDD: 2.5 mg/kg for 12 hours	Third day of therapy: Pre: Just prior to infusion Post: 30 minutes after infusion	ODD: 1.0±0.5 (0 patients >2) TDD: 2.0±1.1 (50% >2)	ODD: 7.9±1.6 (96.3% >5, 3.7% >10) TDD: 6.7±1.1 (92.6% >5, 0 patients >10)	ODD is preferable to TDD for improved SGC and cost savings. All patients passed hearing screens
Vervelde *et al.* (1999) (56)	<38 weeks n=34 No control group	None	3 mg/kg for 24 hours	Pre: 2 hours before dose Post: 30 minutes after infusion	1.4±0.8 (95% <2, 77% <1.5)	5.4±0.9 (87% >4 53% >5, 0 patients >10)	SGC safe and therapeutic
Ohler *et al.* (2000) (65)	n=49 No control group	None Risk factors: -Birth depression -Vasopressor therapy -Decreased renal function -Decreased cardiovascular function	5 mg/kg/dose ≤35 weeks: + risk factors: for 48 hours - risk factors: for 36 hours >35 weeks: + risk factors: for 36 hours - risk factors: for 24 hours	Pre: before 2nd or 3rd dose Post: 2 hours after 2nd or 3rd dose	≤35 weeks: 0.8 (0.2–1.3) >35 weeks: 0.7 (0.1–1.5) 0 patients >2	≤35 weeks 9.1 (7.2–12.1) >35 weeks 11.0 (6.6–16.7) 0 patients <4	SGC effective and safe. Hearing tests (brainstem auditory evoked potentials) passed by all subjects by 2 months of life
Gooding *et al.* (2001) (66)	Group 1: n=249 (23–41 weeks) Group 2: n=48 (27–41 weeks) Group 3: n=155 (23–42 weeks)	None	Group 1: 2.5 mg/kg for 8–24 hours Group 2: 3.5–4 mg/kg for 12–36 hours Group 3: 4 mg/kg for 18–36 hours	Pre: before 3rd dose Post: 1 hour after 3rd dose	Group 1: 73–92% <1.5 Group 2: 73–100% <1.5 Group 3: 66–94% <1.5	Group 1: 19–39% 5–8 Group 2: 56–100% 5–8 Group 3: 54–64% 5–8	SGC safer with EID
Strickland *et al.* (2001) (36)	EID: n=51 (25–42 weeks) MDD: n=53 (27–42 weeks) (historical controls)	Weight <750 g	EID: >2.5 kg: (5 × weight)–1 for 24 hours 1–2.49 kg: 1.5 x [(5 x weight)–1] for 36 hours 0.75–0.99 kg: 2 x [(5 x weight)–1] for 48 hours MDD: 2.5 mg/kg for 8–24 hours	Pre: just before dose Post: 60 minutes after infusion	EID: 0.7±0.6 (97.5% <2) MDD: 1.5±0.68 (51% <2)	EID: 13.1±3.6 (78% >10= target peak) MDD: 7.5±1.5 (87% 6–10)	EID achieves more therapeutic peak and trough SGC than MDD
Agarwal *et al.* (2002) (60)	≥2,500 g ≤7 days of life ODD: n=20 TDD: n=21	-Apgar <5 at 5 minutes -Asphyxia -Shock -CPR -Seizures -Kidney or ear anomalies -Life-threatening congenital anomalies -Neuromuscular disorder	ODD: 4 mg/kg for 24 hours TDD: 2.5 mg/kg for 12 hours	Pre: just before dose Post: 30 minutes after infusion	ODD: 0.9±0.3 (0 patients >2) TDD: 1.6±0.6 (29% >2)	ODD: 8.9±1.5 (100% 6–12) TDD: 6.8±1.1 (71% 6–12)	ODD is safe and efficacious when compared with TDD. No patients failed hearing screens
Avent *et al.* (2002) (67)	BW <1,200 g EID: n=39 MDD: n=13 BW 1,200–2,000 g EID: n=21 MDD: n=13 BW >2,000 g EID: n=19 MDD: n=15 (historical controls)	-Abnormal renal function	Gentamicin or tobramicin <1,200 g EID: 5 mg/kg for 24–48 hours MDD: 2.5 mg/kg for 18–24 hours 1,200–2,000 g EID: 5 mg/kg for 24–36 hours MDD: 2.5 mg/kg for 12–18 hours >2,000 g EID: 5 mg/kg for 24–36 hours MDD: 2.5 mg/kg for 8–12 hours	Pre: just prior to dose Post: 2 hours after infusion	<1,200 g EID: 0.7±0.5 MDD: 1.2±0.74 1,200–2,000 g EID: 0.5±0.31 MDD: .3±0.6 >2,000 g EID: 0.4±0.18 MDD: 1.4±0.61 EID: 0 patients >2 MDD: 14.6% >2	<1,200 g EID: 8.0±1.67 MDD: 5.8±2.16 1,200–2,000 g EID: 8.6±1.26 MDD: 6.0±1.86 2,000 g EID: 8.9±1.88 MDD: 7.6±1.93 EID: 97% >5 MDD: 73.2% >5	SGC more likely in therapeutic range with EID
Rastogi, *et al.* (2002) (37)	600–1,500 g <7 days of life EID: n=30 MDD: n=28	-Outborn infants -Apgar <5 at 5 minutes -Cardiopulmonary arrest -Shock -Seizures -Life-threatening congenital malformations -Kidney or ear anomalies -Neuromuscular disorder	600–1,000 g EID: 5 mg/kg for 48 hours MDD: 2.5 mg/kg for 24 hours 1,001–1,500 g EID: 4.5 mg/kg for 48 hours MDD: 3 mg/kg for 24 hours	Pre: 30 minutes before 48 hour dose Post: 30 minutes after 48-hour dose	EID: 0.70±0.3 MDD: 1.32±0.4	EID: 8.52±2.1 (90% 6–12, 0 patients <5) MDD: 6.51±1.71 (55% 6–12, 18% <5)	SGC more therapeutic with EID. All patients passed hearing tests (brainstem auditory evoked potentials) at follow-up
Hansen *et al.* (2003) (68)	≤7 days of life ODD: n=214 (75 patients <35 weeks) No control group	-Levels not available -Incorrect dose for weight -Multiple levels from same patient (only first set of levels included)	ODD <35 weeks: 3 mg/kg for 24 hours ODD ≥35 weeks: 4 mg/kg for 24 hours	Pre: just prior to 3rd dose Post: 30 minutes after 3rd dose	1.0±0.4 (0 patients >2)	7.8±1.7 (88% 6–12)	SGC safe and effective
Bajaj *et al.* (2004) (69)	EID: n=60 (24–40 weeks) MDD: n=50 (24–40 weeks) (Historical controls)	None	EID: 4 mg/kg for 24–36 hours MDD: 2.5 mg/kg for 12–24 hours	Pre: just prior to 3rd dose Post: 1 hour after 3rd dose	EID: 96.6% <2 MDD: 98% <2	EID: 20% <5 MDD: 92% <5	Peak SGC improved with EID
Mercado *et al.* (2004) (77)	<34 weeks, 750–2,000 g EID: 750–1,500 g n=10 1,501–2,000 g n=9 Control: 750–1,500 g n = 10 1,501–2,000 g n = 11	-Asphyxia and shock; -Vasopressor/diuretic treatment -Congenital or chromosomal abnormalities -Haemodynamically significant patent ductus arteriosus -Mothers received drugs affecting renal function	EID: 750–1,500 g: 4 mg/kg for 48 hours 1,501–2,000 g: 4.5 mg/kg for 48 hours Control: 750–1,500 g: 2.5 mg/kg for 24 hours 1,501–2,000 g: 2.5 mg/kg for 18 hours	Pre: 30 minutes prior to 2nd (EID) or 3rd (control) dose Post: 30 minutes after 2nd (EID) or 3rd (control) dose	EID: 0 >2 Control: 1 >2	EID: 0 <5, 2 >12 Control: 7 patients <5, 0 >12	Peak and trough SGC improved with EID. 2 patients in control group and 1 patient in EID group failed hearing screens
Tugay *et al.* (2006) (76)	Preterm infants (≤37 weeks) n=61 (32.36±3.30 weeks) No control group	-Small for gestational age -Signs of perinatal asphyxia -Moderate to severe respiratory distress syndrome -Hyperbilirubinaemia -Hypotension -Severe cardiac anomalies -Renal malformation -Receiving drugs that could affect renal function	≤29 weeks: 5 mg/kg for 48 hours 30–33 weeks: 4.5 mg/kg for 48 hours 34–37 weeks: 4 mg/kg for 36 hours	Pre: prior to 3rd dose Post: 30 minutes after 3rd dose	8.1% >2	18% >9.99	No significant differences in SCR before and after treatment

BUN=Blood urea nitrogen; CPR=Cardio-pulmonary resuscitation; EID=Extended-interval dosing; IM=Intramuscular; LGA=Large for gestational age; MDD=Multiple-daily dosing; ODD=Once-daily dosing; SGA=Small for gestational age; SCR=Serum creatinine; SGC=Serum gentamicin concentration; TDD=Twice-daily dosing

**Table 3:** Studies of extended-interval dosing of gentamicin in neonates in developing countries

Reference	Population	Exclusions	Dose	Monitoring of levels	Mean trough level (μg/mL)	Mean peak level (μg/mL)	Conclusions
Krishnan *et al.* (1997) (58) India	32–36 weeks ODD: n=9 TDD: n=9	SCR <1 mg/dL	ODD: 4 mg/kg for 24 hours TDD: 2.5 mg/kg for 12 hours	Pre: just before 2^nd^ dose Post: 60 minutes after infusion	ODD: 1.96±0.6 TDD: 2.76±0.7	ODD: 6.56±1.66 (0 patients <4) TDD: 5.45	ODD higher peaks and lower troughs than TDD
Solomon *et al.* (1999) (59) India	ODD: 37 patients TDD: 36 patients Preterm: 32–36 weeks Term: ≥37 weeks	-Haemodynamic instability -Abnormal urine output	ODD: 4 mg/kg for 24 hours TDD: 2.5 mg/kg for 12 hours	Pre: 30 minutes before dose Post: 60 minutes after infusion	ODD Preterm: 1.85±0.86 (30.8% >2) Term: 1.33±1 (8.3% >2) TDD Preterm: 1.98±1.09 (58.3% >2) Term: 1.55±1 (12.5% >2)	ODD Preterm: 7.38±2.29 (84.8% 4–10) Term: 7.1±2.64 (75% 4–10) TDD Preterm: 6.69±2.42 (75.1% 4–10) Term: 6.96±2.83 (75.1% 4–10)	ODD equally efficacious SGC as TDD
Chotigeat *et al.* (2001) (62) Thailand	≥2,000 g ≥34 weeks <7 days of life ODD: n=27 TDD: n=27	-Apgars <4 at 1 minute and/or <6 at 5 minutes -Allergy to aminoglycosides -Congenital anomalies -Renal failure -Neuromuscular disorder	ODD: 4–5 mg/kg for 24 hours TDD: 2–2.5 mg/kg for 12 hours	Pre: 30 minutes before dose Post: 30 minutes after infusion	ODD: 0.9±0.35 (0 patients >2) TDD: 1.44±0.49 (7.4% >2)	ODD: 8.92±1.59 (0 patients <4) TDD: 5.94±1.57 (3.7% <4)	ODD more therapeutic SGC than TDD
Alsaedi *et al.* (2003) (79) Saudi Arabia	≥2,500 g ≤7 days of life ODD: n=50 TDD: n=50 (historical controls)	-Preterm	ODD: 4 mg/kg for 24 hours TDD: 2.5 mg/kg for 12 hours		ODD: 6% >2 TDD: 26% >2	ODD: 8.4±1.8 (98% >5, 58% 8–12) TDD: 6.7±2 (86% >5, 24% 8–12)	ODD more therapeutic and likely safer than TDD
Kosalaraksa *et al.* (2004) (70) Thailand	≥2,000 g ≤7 days of life ODD: n=33 TDD: n=31	-Apgar ≤6 at 5 minutes -Perinatal asphyxia -Shock -Cardio-pulmonary arrest -Seizure -Neuromuscular disorder -Anomalies of kidneys or ears	ODD: 5 mg/kg for 24 hours TDD: 2.5 mg/kg for 12 hours	ODD: Pre: before 4^th^ dose Post: 30 minutes after 3rd dose TDD: Pre: before 7th dose Post: 30 minutes after 6th dose	ODD: 1.6±1.1 (22% >2) TDD: 2.6±1.2 (68% >2)	ODD: 10.1±3.0 (0 patients <4, 21% >12) TDD: 7.8±2.0 (4% <4, 0 patients >12)	ODD more therapeutic SGC than TDD
English *et al.* (2004) (72) Kenya	ODD: n=155 (49% <7 days of life, 4% <1.5 kg, 12% 1.5–2.0 kg) MDD: n=142 (36% <7 days of life, 2% <1.5 kg, 10% 1.5–2.0 kg)	->2–3 months of age -<1 kg -Tetanus -Congenital malformation -Anuria for 24 hours -Elevated SCR	ODD: load with 8 mg/kg, then 2–6 mg/kg for 24 hours MDD: 2.5 mg/kg for 8–12 hours Gentamicin given IM routinely	Pre: prior to second and fourth doses Post: 1 hour after first and third doses	ODD: 0.6 (95% CI 0.3- 1.3) (6% ≥2) MDD: 1.1 (95% CI 0.7- 2.3) (24% ≥2)	ODD: 9.0 (95% CI 8.3–9.9) (12% <4) MDD: 4.7 (95% CI 4.2–5.3) (19% <4)	ODD more therapeutic SGC than MDD
Kiatchoosakun *et al.* (2005) (71) Thailand	≥34 weeks, ≥2,000 g, <7 days of life ODD: n=105 No control group	-Apgar <4 at 1 minute and/or 5 minutes -Shock -Cardiopulmonary arrest -Seizures -Life-threatening congenital malformation -Anomalies of kidneys or ears	ODD: 4 mg/kg for 24 hours	Pre: 30 minutes prior to 3rd dose Post: 30 minutes after 3rd dose	ODD: 0.99±0.57 (93.3% <2)	ODD: 7.33±2.77 (97% > 4, 2.85% >12)	ODD resulted in therapeutic SGC. Also all infants with hearing tests had normal results
Kosalaraksa *et al.* (2004) (70) Thailand	≥2,000 g ≤7 days of life ODD: n=33 TDD: n=31	-Apgar ≤6 at 5 minutes -Perinatal asphyxia -Shock -Cardio-pulmonary arrest -Seizure -Neuromuscular disorder -Anomalies of kidneys or ears	ODD: 5 mg/kg for 24 hours TDD: 2.5 mg/kg for 12 hours	ODD: Pre: before 4^th^ dose Post: 30 minutes after 3rd dose TDD: Pre: before 7th dose Post: 30 minutes after 6th dose	ODD: 1.6±1.1 (22% >2) TDD: 2.6±1.2 (68% >2)	ODD: 10.1±3.0 (0 patients <4, 21% >12) TDD: 7.8±2.0 (4% <4, 0 patients >12)	ODD more therapeutic SGC than TDD
English *et al.* (2004) (72) Kenya	ODD: n=155 (49% <7 days of life, 4% <1.5 kg, 12% 1.5–2.0 kg) MDD: n=142 (36% <7 days of life, 2% <1.5 kg, 10% 1.5–2.0 kg)	->2–3 months of age -<1 kg -Tetanus -Congenital malformation -Anuria for 24 hours -Elevated SCR	ODD: load with 8 mg/kg, then 2–6 mg/kg for 24 hours MDD: 2.5 mg/kg for 8–12 hours Gentamicin given IM routinely	Pre: prior to second and fourth doses Post: 1 hour after first and third doses	ODD: 0.6 (95% CI 0.3- 1.3) (6% ≥2) MDD: 1.1 (95% CI 0.7- 2.3) (24% ≥2)	ODD: 9.0 (95% CI 8.3–9.9) (12% <4) MDD: 4.7 (95% CI 4.2–5.3) (19% <4)	ODD more therapeutic SGC than MDD
Kiatchoosakun *et al.* (2005) (71) Thailand	≥34 weeks, ≥2,000 g, <7 days of life ODD: n=105 No control group	-Apgar <4 at 1 minute and/or 5 minutes -Shock -Cardiopulmonary arrest -Seizures -Life-threatening congenital malformation -Anomalies of kidneys or ears	ODD: 4 mg/kg for 24 hours	Pre: 30 minutes prior to 3rd dose Post: 30 minutes after 3rd dose	ODD: 0.99±0.57 (93.3% <2)	ODD: 7.33±2.77 (97% > 4, 2.85% >12)	ODD resulted in therapeutic SGC. Also all infants with hearing tests had normal results
Darmstadt *et al.* (2007) (78) India, Bangladesh	<3,000 g EID: n=110 No control group	-Major congenital anomalies -Unstable haemodynamic status -Renal compromise	<2,000 g: 10 mg for 48 hours 2,000–2,249 g: 10 mg for 24 hours >2,500 g: 13.5 mg for 24 hours	Pre: 30 minutes prior to 3rd dose (for 24 hours) Post: 1 hour after 3rd dose (for 24 hours) 2 hours prior, and 24 hours after 2nd dose (for 48 hours)	≥2: 14 patients	<4: 1 patient >12: 22 patients	EID safe and effective. No association between abnormal hearing screen or increased SCR with SGC outside therapeutic range

In 2006, a Cochrane review compared once-daily dosing with multiple-daily dosing of gentamicin regimens for the treatment of suspected or proved sepsis in preterm neonates (75). The authors included 11 studies and 574 neonates >32 weeks gestation in which once-daily dosing (but not longer intervals) was compared with multiple-daily dosing regimens among newborns of ≤28 days and included two studies in which intramascular, in addition to intravenous, dosing was used. The studies reviewed reflected both developed (7 studies)- and developing (4 studies)-country settings. In this review, we included 16 additional studies in neonates from developed countries (36,37,54-56,61,63-69,76-77) and four additional studies from developing countries (71,72,78,79), adding significantly to the evidence base for extended-interval dosing. The Cochrane review cited reasons for excluding several studies included in this review as: use of a loading dose (72), non-randomized or quasi-randomized study design (36,61,63,68,79), or comparison of once-daily to longer-dosing intervals (37,77). Among the studies included in our review, two were published after the period of inclusion for the Cochrane review (71,76), three were purely descriptive studies (55,56,65), and five were studies in which either inconsistent dosing schedules were used in the control groups or the dosing regimens tested included ranges of dosing rather than a specific dose (54,64,66,67,69). We included all these studies in this review to examine as much available evidence for the safety of extended-interval dosing of gentamicin as possible.

The primary outcomes of the Cochrane review included clinical efficacy (clearance of sepsis) and pharmacokinetic efficacy (peak serum concentrations ≥5 μg/mL and trough concentrations <2 μg/mL). Secondary outcomes included ototoxicity and nephrotoxicity. The authors concluded that the pharmacokinetic properties of once-daily dosing were superior to multiple-daily dosing regimens in that higher peak levels can be achieved while avoiding potentially toxic trough levels. They suggested that further extending dosing intervals to 36–48 hours might be appropriate. A recent meta-analysis of aminoglycoside dosing in children that included 24 studies in patients aged up to 20 years (including 6 studies in neonates, 10 in developing countries) also reported that extended-interval dosing provided similar efficacy and safety compared to multiple-daily dosing regimens (80).

### Developed-country studies

#### Dosing of 3–4 mg/kg

In developed countries, in studies in which patients were given once-daily dosing of gentamicin at doses ranging from 3 to 4 mg/kg, trough levels almost uniformly were <2 μg/mL (Table 2). In seven studies, no patients had trough levels above 2 μg/mL (51,54,57,60,61,63,77), and in other studies, 5% (56), 18% (76), and 11% (55) of patients had trough levels of >2 μg/mL. Bajaj *et al*. performed an observational study comparing standard-dose gentamicin (2.5 mg/kg administered every 12–24 hours) with an extended-interval dosing regimen (4 mg/kg administered every 24–36 hours) in 110 newborns with gestational ages 24–40 weeks (69). They reported that similar numbers of patients had trough levels of <2 μg/mL for the standard and extended-interval dosing regimens (98% and 96.6% respectively), but a lower number of patients receiving extended-interval dosing compared to the standard-dose regimen had sub-therapeutic peak levels of <5 μg/mL (20% and 92% respectively). Similarly, Hansen *et al*. measured serum gentamicin concentrations in 214 newborns receiving once-daily dosing (3 mg/kg for patients <35 weeks gestation and 4 mg/kg for those ≥35 weeks) and reported that peak values of 6–12 μg/mL were achieved in 88% of patients and trough values of >2 μg/mL were noted in no patients studied (68). Other investigators have noted similar improved therapeutic levels in neonates with once-daily dosing compared to multiple-daily dosing (66).

#### Dosing >4 mg/kg

Published studies of particular interest for this review are those in which doses of >4 mg/kg were given by extended-interval dosing. Such dosing regimens may be particularly applicable for use in developing countries, as this would create the possibility of giving the same dose at different intervals to neonates in different weight categories (e.g. a longer-dosing interval for patients of <2,000 g) while achieving therapeutic antibiotic serum levels.

Hayani *et al*. compared twice-daily dosing of 2.5 mg/kg gentamicin (n=15) with once-daily dosing of 5 mg/kg (n=11) in neonates >34 weeks gestation (range: 35–41 weeks) (26). All infants in the once-daily dosing group had therapeutic peak gentamicin concentrations while two patients in the twice-daily dosing group had sub-therapeutic peak concentrations <4 μg/mL. Seven patients had trough concentrations >2 μg/mL. One was a preterm infant in the once-daily dosing group, and six were term infants in the twice-daily dosing group. No nephrotoxic effects were observed.

De Alba Romero *et al*. compared twice-daily dosing of 2.5 mg/kg of gentamicin (n=32) with once-daily dosing of 5 mg/kg/d (n=33) in infants of ≥1,200 g birthweight (53). Both premature and term infants were included in the study. All infants in the once-daily dosing group had higher therapeutic peak concentrations (9.5 μg/mL±1.7 vs 6.4 μg/mL±1.6) and lower trough concentrations (1.4 μg/mL±0.7 vs 2.2 μg/mL±1.0) than those in the control group. Only 3% of once-daily dosing patients—all preterm infants—had a high trough level; no term infants required the adjustment of dosage. There was no difference in clinical outcome between the two groups. In addition, once-daily dosing of gentamicin had practical advantages (less nursing time spent giving medication). The authors concluded that with once-daily dosing, serum drug levels were more favourable for achieving both efficacy and reduced toxicity compared to twice-daily dosing.

In another study, patients weighing 600–1,000 g were given gentamicin of either 5 mg/kg every 48 hours or 3 mg/kg every 24 hours while those weighing 1,001–1,500 g were given either 4.5 mg/kg every 48 hours or 2.5 mg/kg every 24 hours (37). Trough concentrations were significantly lower in the higher dose, 48-hour interval dosing groups, and peak concentrations were in the therapeutic range significantly more often (90% v 55%). These authors suggested that the 48-hour dosing interval with the doses used might be too conservative and indicated that a shorter dosing interval of 36 hours might be optimal. These data suggest that, in very low-birthweight infants, extended-interval dosing may perform best if extended beyond 24 hours.

In a particularly instructive study by Stickland *et al*., modelling of gentamicin levels in neonates led to the development of the following optimized dosing schedule that then was tested prospectively: neonates weighing >2,500 g received a dose of (5 × weight in kg) every 24 hours, and those weighing 1,000–2,499 g received a dose of 1.5 x [(5 x weight)-1] every 36 hours (36). Thus, for example, a baby weighing 1.5 kg received doses of 9.75 mg (6.5 mg/kg) every 36 hours, and a baby weighing 2.0 kg received doses of 13.5 mg (6.75 mg/kg) every 36 hours. In this study, only one patient (2%) had a trough value of >2 μg/mL.

Tugay *et al*. evaluated the acute effects of extended-interval dosing of gentamicin on glomerular and tubular renal functions among 61 preterm neonates with suspected sepsis. A dose of 5 mg/kg was administered every 48 hours for patients ≤29 weeks gestational age, 4.5 mg/kg was administered every 48 hours for patients 30–33 weeks gestational age, and 4 mg/kg was administered every 36 hours for patients 34–37 weeks gestation (76). Serum peak and trough levels, serum and urine creatinine, sodium and potassium levels, and urine albumin and calcium levels were measured at baseline, after the third dose of gentamicin, and at 48–72 hours after completion of gentamicin therapy. Overall, high trough (≥2 mg/L) and peak (≥9.99 mg/L) levels were found in 5 (8.1%) and 11 (18%) neonates respectively. Additionally, 11 (18%) patients had sub-therapeutic peak levels (<6.1 mg/L). Of note, none of the neonates included in this study had proven sepsis during the study period. Although the authors reported weak but positive correlations between trough and peak gentamicin levels with serum creatinine, urine albumin/creatinine ratio, fractional excretion of potassium, fractional excretion of sodium, and urinary calcium/creatinine ratios, pretreatment, treatment, and post-treatment serum creatinine and fractional excretion of potassium values did not show any statistically significant difference for sub-therapeutic (peak <6.1 mg/L), therapeutic (trough <2 mg/L, peak 6.1–9.99 mg/L) and high (trough >2 mg/L, peak >9.99 mg/L) trough and peak gentamicin levels. These data suggest that the extended-interval regimens were safe.

#### Unpublished data

In addition to published data, a co-author (MMB) of this review conducted a study in 1999 of once-daily dosing of gentamicin for neonates in the Neonatal Intensive Care Unit (NICU) at Duke University Hospital. The once-daily dosing protocol implemented for patients without renal dysfunction was as follows: infants ≤29 weeks gestation received 3.5–4 mg/kg/dose every 48 hours, and infants ≥30 weeks gestation received 3.5–4 mg/kg/dose every 24 hours. Renal dysfunction was defined as serum creatinine of >1.7 μg/mL. Patients with renal dysfunction were given 2.5 mg/kg of gentamicin for one dose, and a pharmacy consultant provided recommendations for further dosing based on evaluation of pharmacokinetic parameters.

During the study period, gentamicin levels were not monitored for patients who received therapy for ≤48 hours. If the decision was made to continue therapy beyond 48 hours, gentamicin levels were monitored around the second dose for patients receiving gentamicin every 48 hours and around the third dose for patients receiving gentamicin every 24 hours. The goal of therapy was a trough level of <1.5 μg/mL and a peak level of 5–10 μg/mL. In total, 244 courses of gentamicin therapy were administered during the study period. With these dosage and patient-management regimens, adjustment of dosage was required for 26 (10.7%) of the 244 courses of therapy. Potentially toxic drug levels (i.e. trough >2 μg/mL or peak >10 μg/mL) were found in 12 (5%) cases. Based on these results, the once-daily dosing gentamicin protocol has been used in the NICU at Duke University since the trial, with good results.

#### Developing-country studies

Studies on extended-interval dosing among neonates in developing countries are summarized in Table 3. We evaluated these studies separately, given the higher rates of low birthweight and malnutrition and the greater likelihood of sub-therapeutic hydration status of infants in these settings. Moreover, the focus of this review was to identify optimal extended-interval dosing regimens for infants in low-resource settings. Although these differences between settings could potentially result in differing pharmacokinetic profiles among target populations, at this time there are insufficient data to explore this specific question.

In India, once-daily dosing of preterm infants between 32–36 weeks gestational age with 4 mg/kg of gentamicin (n=9 patients) was compared with twice-daily dosing with 2.5 mg/kg/dose (n=9 patients) (58). Therapeutic peak levels of gentamicin after the first dose were achieved only with the once-daily dosing regimen, and the mean peak levels at steady state were not significantly different for the two regimens. Patients who received 4 mg/kg once daily had a mean trough level of 1.96±0.60 μg/mL, whereas those who received the twice-daily dosing regimen had trough levels of 2.76±0.70 (p=0.019). It was concluded that once-daily dosing of gentamicin in preterm infants (32–36 weeks) provides initial peak serum concentrations above the MIC of gram-negative bacteria and trough concentrations below potentially toxic levels. Further, the recommendation was made to switch to once-daily dosing due to its monetary, logistical and pharmacokinetic advantages over twice-daily dosing.

In another study conducted in India, 73 newborns were stratified by gestational age (32–36 weeks gestational age, and term infants) and randomized to receive either once-daily dosing (n=37) of 4 mg/kg or twice-daily dosing (n=36) of 2.5 mg/kg/dose (59). The authors reported that both mean peak and trough concentrations were similar for the two study groups, and they concluded that once-daily dosing was as effective as twice-daily dosing and was more cost-effective.

In Thailand, Chotigeat *et al*. treated 54 infants of >2,000 g and ≥34 weeks gestational age, within the first seven days of life, with gentamicin given by either once-daily dosing (4–5 mg/kg) or multiple-daily dosing (2–2.5 mg/kg every 12 hours) (62). The authors reported that, while three patients in the multiple-daily dosing group had peak or trough levels outside the acceptable ranges of peak 4–12 μg/mL, trough <2 μg/mL, all patients in the once-daily dosing group had acceptable peak and trough values. In an additional study in Thailand, 36 newborns received either once-daily dosing (5 mg/kg) or twice-daily dosing (2.5 mg/kg) of gentamicin; once-daily dosing resulted in a lower proportion of patients with potentially toxic trough levels of ≥2 μg/mL compared to twice-daily dosing (22% vs 68% respectively), while the proportion of patients with therapeutic peak levels of ≥4 was similar between the two groups (100% and 96% respectively) (70). Kiatchoosakum *et al*. administered gentamicin by once-daily dosing (4 mg/kg) to 105 neonates in Thailand who were ≥2,000 g and ≥34 weeks gestational age; 97% had peak levels of >4 μg/mL, and 93% had trough levels of <2 μg/mL (71). No abnormal hearing test results (audiometry) were observed among 100 of these patients prior to discharge, and none of the 47 patients had abnormal brainstem auditory evoked potentials at a six-month follow-up visit. They reported no instances of nephrotoxicity.

In Kenya, English *et al*. conducted a study comparing once-daily dosing (2–6 mg/kg) after an 8-mg/kg loading dose with multiple-daily dosing (2.5 mg/kg given 2–3 times daily) of gentamicin (72). In this study, gentamicin was routinely administered as an intramascular injection. The authors reported that the serum trough concentrations were potentially toxic (≥2 μg/mL) in 6% and 24% of patients receiving once-daily dosing and multiple-daily dosing respectively, suggesting that once-daily dosing was safer than multiple-daily dosing. Peak serum gentamicin concentrations were potentially sub-therapeutic (<4 μg/mL) in 12% and 19% of patients receiving once-daily dosing and multiple-daily dosing respectively.

In a study of extended-interval dosing of gentamicin among neonates hospitalized for suspected sepsis in India and Bangladesh, Darmstadt *et al*. reported on the pharmacokinetics of a simplified weight-based gentamicin dosing regimen (78). In total, 110 patients whose birthweights were ≤3,000 g were included in this study. The dose of gentamicin administered intravenously varied by birthweight category: 10 mg (absolute dose, not mg/kg) every 48 hours for neonates of <2,000 g; 10 mg every 24 hours for neonates of 2,000–2,249 g; and 13.5 mg every 24 hours for neonates of ≥2,500 g. The authors found that pharmacokinetic parame-ters, i.e. elimination rate constant, serum half-life, and volume of distribution were similar among patients from both centres. Only one patient had a peak concentration <4 μg/mL, and 20% of patients had peak concentrations >10 μg/mL. 12.7% of patients had a trough gentamicin concentration >2 μg/mL. However, no patients with high trough and/or peak values showed a significant increase in serum creatinine. In total, 76 patients had hearing testing performed at follow-up, of which only two had abnormal findings, but neither of these patients had elevated gentamicin peak or trough concentrations. The authors concluded that their extended-interval dosing regimen can be used safely and effectively in the treatment of neonatal sepsis in low-resource settings.

Of the studies included in this review, information regarding potential ototoxicity with extended-interval dosing of gentamicin regimens was available for 10 studies (37,51,53,60,63,65,71,75,77,78). Although none of these studies was powered to demonstrate a significant difference with respect to hearing loss, the results of these studies suggest that there was no appreciable difference in ototoxicity between neonates receiving extended-interval dosing compared to multiple-daily dosing regimens of gentamicin (Table 2-3).

## DISCUSSION

Review of available data suggests that gentamicin therapy in neonates in developed- and developing-country studies using extended-interval dosing with intervals of ≥24 hours and doses of ≥4 mg/kg has reduced potential for toxicity while conferring equal or greater clinical efficacy; therefore, its use, in combination with a penicillin derivative for gram-positive coverage, is growing as the standard for treatment of neonates in both developed and developing countries (25,48,81). None of the clinical studies included in this review was powered individually to show a statistically significant difference in clinically-relevant outcomes of treatment success such as microbiological cure or decreased mortality; rather, the experimental designs rely on the assumption that the surrogate endpoint of gentamicin peak serum concentration accurately reflects these outcomes. However, given that pooled data, such as those presented in the Cochrane Review, suggest equivalence between extended-interval dosing and multiple-daily dosing regimens, these studies provide important evidence suggesting therapeutic efficacy of extended-interval dosing that extends to developing-country settings (75). We found no apparent differences in pharmacokinetics of gentamicin in developed-country settings compared to developing-country settings; however, further analyses are warranted as additional data become available.

While extended-interval dosing does not appear to increase nephrotoxicity or ototoxicity, these outcomes have not been systematically or consistently evaluated in the studies of extended-interval dosing with gentamicin among neonates. Extended-interval dosing is potentially more cost-effective for treating neonatal patients with gentamicin, resulting in decreased time for the pharmacy and nursing staff, fewer administered doses per day, decreased costs of drug supply, and reduced costs of therapeutic drug monitoring. Furthermore, it is possible that extended-interval dosing is less anxiety-provoking than multiple-daily dosing to patients and their families. This may, in fact, lead to improved acceptability of treatment and adherence to treatment in these settings as was observed in a recent study of hospital-based treatment of pneumonia in children comparing intramuscular once-daily dosing with multiple-daily dosing of gentamicin from Bangladesh (82). Based on the findings of this review, the development of optimal extended-interval dosing regimens for use in developing countries should be a research priority.

Most hospitals in developing countries do not have the capacity to perform therapeutic drug monitoring for gentamicin. Moreover, in settings where monitoring is available, the associated costs may be prohibitively high. Where gestational age is not consistently and reliably available, weight-based algorithms may be of particular benefit. Treatment regimens could be simplified to include a limited selection of dosages (i.e. likely no more than two) but varying intervals based on weight. The dose in mg/kg that would be administered to newborns of various weights with two different unit doses (10 mg and 13.5 mg) is illustrated in the Figure. The weight range illustrated represents birthweights obtained from a rural North Indian cohort which ranged from 2,000 g (5^th^ percentile) to 3,500 g (95^th^ percentile) (83). For patients in the 2,000–2,499 g strata, for example, the dosage administered would vary from 4 to 5 mg/kg. Results of our review suggest that a 10-mg dose could be administered safely and effectively at two different intervals, i.e. every 24 hours or every 48 hours, to neonates in two different, corresponding weight categories, e.g. 2,000–2,499 g and <2,000 g respectively. Unit doses could potentially be prepared, for example, in pre-filled syringes, such as the Uniject™ (84) device, further reducing the risk of medication-administration errors, enhancing ease of use (potentially even by community-based health workers), and potentially creating a more favourable cost-benefit profile. Through such a simplified dosing scheme, it is conceivable that, in some settings where facility-based or clinic-based care is unavailable or inaccessible, but where community case management of serious infections exists, doses could be administered in the home, where the majority of births in many low-resource settings occur, at the earliest possible point in the presentation of clinical sepsis. By treating uncomplicated cases of presumed neonatal sepsis in the home or at community-based clinics, the potential burden to the family of accessing hospital care and the costs associated with hospitalizations could also be alleviated. However, even in developing-country settings where home-based or clinic-based management of serious infections with parenteral antibiotics is not acceptable or feasible, such an approach could facilitate life-saving treatment in health facilities at various levels of the health system, extending from peripheral, primary-care centres to referral hospitals. Further research is needed to explore these potential applications of extended-interval dosing for gentamicin in various developing-country settings.

**Fig.**
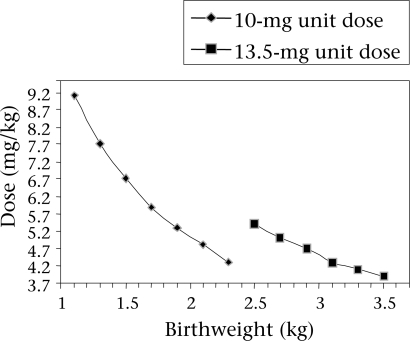
Dose ranges in mg/kg using 10- and 13.5-mg unit doses by birthweight

In summary, extended-interval dosing of gentamicin provides for a more favourable pharmacokinetic profile the multiple-daily dosing, the potential for enhanced clinical efficacy, reduced risk for emergence of resistance organisms, and decreased toxicity at reduced cost. Although data are limited on anticipated effects of once-daily doses of gentamicin in the range of 5 mg/kg, it appears that the risk of a 5-mg/kg dose is comparable with that for a 4-mg/kg dose. Based on the findings of this review, with the aim of developing a simple extended-interval dosing regimen for use of gentamicin in neonates in developing-country settings with presumed sepsis or culture-proven sepsis with a gentamicin-susceptible pathogen, we propose the following dosing regimen (absolute doses, not mg/kg):

- 13.5 mg every 24 hours for neonates of ≥2,500 g
- 10 mg every 24 hours for neonates of 2,000–2,499 g
- 10 mg every 48 hours for neonates of <2,000 g

This dosage schedule has been successfully implemented in a prospective study of hospitalized neonates in Bangladesh and India (78).
